# Measured vs estimated resting energy expenditure in children and adolescents with obesity

**DOI:** 10.1038/s41598-023-40435-8

**Published:** 2023-08-14

**Authors:** Sofia Tamini, Diana Caroli, Adele Bondesan, Laura Abbruzzese, Alessandro Sartorio

**Affiliations:** 1 Istituto Auxologico Italiano, IRCCS, Experimental Laboratory for Auxo-Endocrinological Research, Piancavallo-Verbania, Italy; 2 Division of Rehabilitative Medicine, Istituto Auxologico Italiano, IRCCS, Piancavallo-Verbania, Italy; 3 Istituto Auxologico Italiano, IRCCS, Experimental Laboratory for Auxo-Endocrinological Research, Milan, Italy

**Keywords:** Nutrition disorders, Obesity, Obesity

## Abstract

Pediatric obesity requires early targeted interventions consisting mainly of a low-calorie diet prescribed based on resting energy expenditure (REE), often estimated through predictive equations. The aim of this study was to define the prevalence of "hypo-", "normo-" and "hypermetabolic" in a large cohort of children and adolescents with obesity by comparing measured and estimated REE and to evaluate the characteristics related to these metabolic statuses in both males and females. The study population was divided into the three subgroups by comparing REE measured using indirect calorimetry and estimated using the Molnar equation, and subsequently analyzed. The majority of the participants (60.6%) were normometabolic, 25.5% hypermetabolic and 13.9% hypometabolic. No significant differences in age, Tanner stage, systolic blood pressure, or the presence of metabolic syndrome were found. However, the hypermetabolic subgroup was significantly lighter, shorter, with lower hip and waist circumferences, had a greater amount of fat-free mass and lower fat mass, significantly lower diastolic blood pressure, and a significantly higher frequency of non-alcoholic liver steatosis. Pediatric obesity is more associated with normal or increased REE than with a hypometabolic condition, suggesting that estimation of energy expenditure with predictive equations is still inadequate for prescribing the appropriate diet plan.

## Introduction

Obesity prevalence, especially in the pediatric population, has dramatically increased over the last decade, becoming one of the major health concerns worldwide^1^. Childhood obesity determines an increased risk for the development of non-communicable diseases in adulthood, such as type 2 diabetes mellitus, cardiovascular, gastrointestinal, and non-alcoholic fatty liver diseases, metabolic syndrome, and different types of cancer^1,2^. Moreover, children with obesity are very likely to become adults with obesity, with more critical health issues than those who develop obesity in adulthood^1,3,4^. Environmental changes, particularly easy access to high-calorie, energy-dense, low in nutrients and low-quality foods, increased consumption of sugary beverages, and sedentary lifestyles, are strictly linked with this phenomenon. In addition, the COVID-19 pandemic has aggravated the epidemic of childhood obesity, leading to significant weight gain in schoolchildren and adolescents by creating an even more obesogenic and more sedentary environment^5^. For this reason, it appears clear that early, targeted, and effective interventions are essential to counteract the disease and promote a healthy lifestyle^6,7^.

The treatment of obesity is challenging and requires a multidisciplinary approach which must be age-appropriate and include diet, physical activity, and behavioral therapy^8^. This is necessary to reduce body weight and correct both wrong habits and sedentary lifestyle^9,10^. A low-calorie diet remains a fundamental part of the obesity management interventions. Therefore, it is essential to prescribe an adequate diet plan, personalized and specific for the growth phase, in order to achieve appropriate and gradual weight loss and to determine improvements in body composition, metabolic parameters, and quality of life^11–13^.

In clinical practice, the first step in determining a tailored diet for each subject is to evaluate their individual energy needs and, consequently, obtain an adequate and commensurate caloric deficit. Hence, accurately identifying the real total energy expenditure (TEE) of every distinct patient is a crucial phase, as it allows quantifying the actual energy requirement and prescribing the appropriate diet plan accordingly^14^.

By definition, TEE is the amount of energy consumed in 24 h by an individual and is given by the sum of three elements: diet-induced thermogenesis (TID), basal metabolism or resting energy expenditure (REE), and energy consumption due to physical activity^14^. TID is a relatively constant parameter among individuals and contributes marginally to TEE, with a maximum of 10%, while the predominant components are REE, defined as the minimum level of energy needed to support vital functions, and physical activity. Physical activity is the most variable parameter, as it is strictly dependent on the lifestyle and planned activities of each person, and for this reason, it exhibits significant variability within and between individuals^14–16^.

The pediatric population might have an additional element to these three major components of energy expenditure. In fact, children and adolescents might require supplementary energy for growth, although in general it can be considered negligible except within the initial few months of life^14^.

As previously stated, individuals who suffer from obesity tend to engage in sedentary lifestyles, with very limited physical activity. This situation is exacerbated in current and modern times, where a busy school schedule, a lack of time for sports, and more sedentary behaviors, such as video games and computer use, can limit physical exercise^14,17,18^. For this reason, taking all this into account, it appears clear that REE is the major component of the TEE in this population, and its correct determination can allow to accurately quantify the energy necessary to optimize weight loss and its maintenance.

The gold standard method to measure REE is indirect calorimetry (IC)^19^, though it has some limitations since it is expensive, time-consuming, not available in every medical structure, and not applicable in ambulatory clinical practice since it is relatively expensive, requires expert technicians, specific instruments, and periodic calibration^20^.

Consequently, as an alternative approach, REE is usually estimated from prediction equations developed using IC as the reference method^21^. Over time, various predictive equations have been validated for the adult population, taking into account different parameters of the subject, such as sex, age, height, weight, and in some cases, even body composition. Those equations attempted to be population-specific and considered the obese status of the individual^22^.

Since it is not possible to use the same equations as the adults in the pediatric population, specific equations have been developed for the younger subjects.

Predictive equations for obesity, however, are not always accurate, and their use is still debated since their accuracy tends to decrease with increasing body weight^14,20,22^. Several validation studies have suggested that the Mifflin-St. Jeor equation is the best predictive equation for the adult population with obesity^20,23,24^, while the Molnar equation is the most accurate for REE estimation, regardless of sex and obesity degree^21,25^, in the pediatric population with obesity.

Recently, our group has conducted a comparison between the estimated REE (eREE), calculated using the Mifflin-St. Jeor equation, and the measured REE (mREE) using IC, in the adult population with obesity to evaluate the prevalence of individuals with reduced metabolism, i.e., a mREE lower than expected. Through this comparison, it was possible to identify the prevalence of participants with obesity who were “hypo-”, “normo-” and “hypermetabolic”. These different metabolic conditions are defined based on the ratio of mREE to eREE; in particular, hypometabolism occurs when the mREE is less than 90% of eREE; the normometabolic condition when the mREE is between 90 and 110% of the eREE and lastly, hypermetabolism when the mREE is greater than 110% of eREE^26^. Contrary to the common belief that obesity is due to a reduced REE, only 8% of the adult study population were hypometabolic^26^.

Taking into account the above considerations, the primary aim of this retrospective study, conducted on a large cohort of children and adolescents with obesity, is to identify the prevalence of "hypo-", "normo-" and "hypermetabolism" in this pediatric population by comparing the estimated eREE (using the Molnar equation) with the measured mREE with IC. The secondary aims are to characterize the three subgroups identified, also describe the differences between males and females, and evaluate how the main anthropometric and clinical characteristics relate to metabolic status.

## Results

The whole study group (n.: 1400, F/M: 807/593, age: 14.3 ± 1.8 years, BMI: 36.7 ± 6.0 kg/m^2^) was divided into three subgroups based on their mREE/eREE percentage ratio: i. hypometabolic: mREE < 90% of eREE; ii. normometabolic: mREE between 90—110% of eREE; iii. hypermetabolic: mREE > 110% of eREE.

The majority of the study population was normometabolic (60.6%), while 25.5% were hypermetabolic and 13.9% were hypometabolic.

The main anthropometric and clinical characteristics of the subgroups are shown in Table 1.

**Table 1:** Anthropometric and clinical characteristics of the three subgroups.

	Hypometabolic (< 90%)	Normometabolic (90%—110%)	Hypermetabolic (> 110%)	*p-*value	*p-*value Hypo vs. Normo	*p-*value Hypo vs. Hyper	*p-*value Hyper vs. Normo
n	194 (13.9%)	849 (60.6%)	357 (25.5%)				
Sex (F/M)	120 (61.9%)/74 (38.1%)	470 (55.4%)/379 (44.6%)	217 (60.8%)/140 (39.2%)	ns^a^	ns^b^	ns^b^	ns^b^
Age (yrs)	14.4 ± 1.9	14.3 ± 1.8	14.2 ± 1.9	ns^d^	ns^c^	ns^c^	ns^c^
Tanner stage	3.8 ± 1.3	3.7 ± 1.3	3.6 ± 1.3	ns^d^	ns^c^	ns^c^	ns^c^
mREE (kcal/day)	1626.8 ± 293.6°	1919.8 ± 321.3	2169.7 ± 388.6°	**^d^	**^c^	**^c^	**^c^
eREE Molnar (kcal/day)	1920.4 ± 333.0	1925.4 ± 319.9	1824.2 ± 294.9	**^d^	ns^c^	**^c^	**^c^
WC (cm)	112.8 ± 15.3	112.6 ± 14.5	108.5 ± 13.4	*^d^	ns^c^	**^c^	**^c^
HC (cm)	121.0 ± 12.7	119.9 ± 12.2	116.1 ± 10.2	**^d^	ns^c^	**^c^	**^c^
BW (kg)	99.6 ± 22.6	99.2 ± 22.0	92.7 ± 19.0	**^d^	ns^c^	**^c^	**^c^
Height (cm)	162.9 ± 10.1	163.0 ± 9.8	160.7 ± 9.2	**^d^	ns^c^	**^c^	**^c^
BMI (kg/m^2^)	37.2 ± 6.2	37.1 ± 6.1	35.4 ± 5.4	**^d^	ns^c^	**^c^	**^c^
mREE/BW (Kcal/kg)	16.6 ± 2.0	19.7 ± 2.2	23.7 ± 3.0	**^d^	**^c^	**^c^	**^c^
FM (kg)	54.0 ± 16.2	52.1 ± 15.8	47.0 ± 12.9	**^d^	ns^c^	**^c^	**^c^
FFM (%)	46.5 ± 5.9	48.2 ± 6.5	49.7 ± 6.0	**^d^	**^c^	**^c^	**^c^
SBP (mmHg)	123.7 ± 11.9	124.3 ± 12.2	123.2 ± 12.0	ns^d^	ns^c^	ns^c^	ns^c^
DBP (mmHg)	78.1 ± 7.0	77.7 ± 7.8	76.4 ± 8.0	**^d^	ns^c^	*^c^	**^c^
MetS (Y/N)	44 (22.7%)/150 (77.3%)	197 (23.2%)/652 (76.8%)	73 (20.4%)/284 (79.6%)	ns^a^	ns^b^	ns^b^	ns^b^
NAFLD (Y/N)	58 (29.9%)/136 (70.1%)	322 (37.9%)/527 (62.1%)	148 (41.5%)/209 (58.5%)	*^a^	*^b^	**^b^	ns^b^

*mREE* measured Resting Energy Expenditure, *eREE* estimated Resting Energy Expenditure, *WC* waist circumference; *HC* hips circumference, *BW* body weight, *BMI* body mass index, *FM* fat mass, *FFM* fat-free mass, *SBP* systolic blood pressure, *DBP* diastolic blood pressure, *MetS* metabolic syndrome, *NAFLD* non-alcoholic fatty liver disease, *ns* non-significant.

*p < 0.05; **p < 0.01; °*p* < 0.01 compared to the eREE value (t-Student test).

^a^Chi-squared test.

^b^Fisher’s exact test.

^c^T-Student test.

^d^ANOVA.

No significant differences were found in terms of sex, age, Tanner stage, systolic blood pressure and presence of MetS between the three subgroups.

By contrast, the hypermetabolic subgroup was significantly lighter (lower BW and BMI), shorter and with lower hips and waist circumferences (*p* < 0.01 for all parameters, except waist circumference with *p* < 0.05) as compared to the other two subgroups, while these characteristics were comparable in the normometabolic and hypometabolic groups.

The analysis of the body composition showed that the hypermetabolic subgroup had the best body composition profile, with a greater amount of fat-free mass and lower fat mass (*p* < 0.01), while the hypometabolic subgroup showed the lowest FFM (*p* < 0.01) even though the amount of FM was comparable to those recorded in normometabolic and hypometabolic subgroups.

The hypermetabolic group had a significantly lower diastolic blood pressure (*p* < 0.05) and a significantly higher frequency of NAFLD when compared to the hypometabolic subgroup (*p* < 0.01), since the hypometabolic had the lowest frequency (*p* < 0.05).

While no significant difference was found in the mREE and the eREE in the normometabolic group, the mREE of hypometabolic participants was significantly lower, by approx. 293 kcal, than eREE (p < 0.01) and the mREE of hypermetabolic significantly higher, of approx. 345 kcal (p < 0.01). Moreover, adjusting the mREE for body weight, the hypermetabolic group showed the greatest energy expenditure per kg of body weight compared to the other two subgroups (*p* < 0.01), while the hypometabolic group had the lowest (*p* < 0.01).

Table 2 summarized the comparison of the same anthropometric and clinical characteristics of the three subgroups when further divided into males and females.

**Table 2:** Anthropometric and clinical characteristics of the three subgroups divided into males and females.

	Hypometabolic			Normometabolic			Hypermetabolic			*p*-value	*p*-value
	F	M	*p-value*	F	M	*p-* value	F	M	*p-* value	F	M
n	120 (61.9%)	74 (38.1%)		470 (55.4%)	379 (44.6%)		217 (60.8%)	140 (39.2%)			
Age (yrs)	14.3 ± 1.9	14.5 ± 1.9	ns^a^	14.4 ± 1.7	14.4 ± 2.0	ns^a^	14.4 ± 1.9	14.0 ± 1.8	ns^a^	ns^c^	*^c^
Tanner stage	3.9 ± 1.3	3.6 ± 1.3	ns^a^	4.1 ± 1.2	3.4 ± 1.3	**^a^	4.0 ± 1.3	3.1 ± 1.2	**^a^	ns^c^	**^c^
mREE (kcal/day)	1501.6 ± 207.6°	1829.8 ± 300.2°	**^a^	1794.5 ± 244.9	2075.1 ± 337.0	**^a^	1997.8 ± 272.7°	2436.2 ± 391.9°	**^a^	**^c^	**^c^
eREE Molnar (kcal/day)	1776.3 ± 218.9	2154.1 ± 354.2	**^a^	1795.3 ± 235.7	2086.8 ± 336.8	**^a^	1685.6 ± 190.7	2039.0 ± 299.6	**^a^	**^c^	ns^c^
WC (cm)	109.4 ± 13.9	118.2 ± 16.0	**^a^	110.2 ± 14.2	115.5 ± 14.3	**^a^	105.3 ± 12.4	113.6 ± 13.4	**^a^	**^c^	ns^c^
HC (cm)	121.3 ± 11.8	120.5 ± 14.1	ns^a^	121.2 ± 11.8	118.4 ± 12.6	**^a^	116.3 ± 9.8	115.8 ± 10.8	ns^a^	**^c^	*^c^
BW (kg)	94.7 ± 18.4	107.7 ± 26.4	**^a^	96.5 ± 19.3	102.5 ± 24.5	**^a^	88.5 ± 15.6	99.1 ± 22.0	**^a^	**^c^	*^c^
Height (cm)	160.0 ± 8.5	167.7 ± 10.8	**^a^	160.45 ± 7.1	166.0 ± 11.6	**^a^	158.2 ± 7.4	164.7 ± 10.2	**^a^	**^c^	ns^c^
BMI (kg/m^2^)	36.8 ± 5.8	37.9 ± 6.9	ns^a^	37.3 ± 6.1	36.8 ± 6.1	ns^a^	35.3 ± 5.2	36.2 ± 5.7	ns^a^	**^c^	ns^c^
mREE/BW (Kcal/kg)	16.1 ± 1.8	17.4 ± 2.0	**^a^	18.9 ± 2.0	20.7 ± 2.1	**^a^	22.9 ± 2.8	25.0 ± 2.9	**^a^	**^c^	**^c^
FM (kg)	51.9 ± 14.1	57.4 ± 18.8	* ^a^	52.3 ± 15.0	51.8 ± 16.7	ns^a^	45.9 ± 11.6	48.7 ± 14.4	*^a^	**^c^	**^c^
FFM (%)	45.8 ± 5.8	47.5 ± 6.0	ns^a^	46.6 ± 5.8	50.1 ± 6.7	**^a^	48.6 ± 5.5	51.3 ± 6.4	**^a^	**^c^	**^c^
SBP (mmHg)	121.4 ± 10.9	127.5 ± 12.5	ns^a^	122.9 ± 11.9	125.9 ± 12.4	**^a^	121.1 ± 10.4	126.6 ± 13.4	**^a^	ns^c^	ns^c^
DBP (mmHg)	77.6 ± 6.7	78.9 ± 7.4	ns^a^	77.5 ± 7.5	78.1 ± 8.1	ns^a^	75.5 ± 7.7	77.6 ± 8.3	* ^a^	**^c^	ns^c^
MetS (Y/N)	15 (12.5%) /	29 (39.2%)/	**^b^	197 (23.2%)/	99 (26.1%) /	ns^b^	34 (15.7%) /	39 (27.9%) /	**^b^	**^d^	ns^d^
	105 (87.5%)	45 (60.8%)		652 (76.8%)	280 (73.9%)		183 (84.3%)	101 (72.1%)			
NAFLD (Y/N)	26 (21.7%) /	32 (43.2%) /	**^b^	129 (27.4%)/	193 (50.9%) /	**^b^	71 (32.7%) /	77 (55%) /	**^b^	ns^d^	ns^d^
	94 (78.3%)	42 (56.8%)		341 (72.6%)	186 (49.1%)		146 (67.3%)	63 (45%)			

*M* males, *F* females, *mREE* measured Resting Energy Expenditure, *eREE* estimated Resting Energy Expenditure, *WC* waist circumference, *HC* hips circumference, *BW* body weight, *BMI* body mass index, *FM* fat mass, *FFM* fat-free mass, *SBP* systolic blood pressure, *DBP* diastolic blood pressure, *MetS* metabolic syndrome, *NAFLD* non-alcoholic fatty liver disease, *ns* non-significant.

*p < 0.05; **p < 0.01; °*p* < 0.01 compared to the eREE value (t-Student test).

^a^T-Student test.

^b^Fisher’s exact test.

^c^ANOVA.

^d^Chi-Squared test.

In the hypometabolic subgroup, no significant differences were found in terms of age, Tanner stage, hip circumference, BMI, FFM, systolic and diastolic blood pressure between males and females. However, hypometabolic females showed significantly lower waist circumference, BW, height, frequency of both MetS and NAFLD (*p* < 0.01) and FM (*p* < 0.05).

The mREE of hypometabolic females was also significantly lower than that of hypometabolic males, of approx. 328 kcal. Moreover, for hypometabolic females, the mREE was significantly lower than eREE (*p* < 0.01) of approx. 275 kcal, and for hypometabolic males of approx. 324 kcal (*p* < 0.01).

Also, in the case of mREE adjusted for body weight, males showed the greatest energy expenditure per kg of body weight compared to females (*p* < 0.01).

In the normometabolic subgroup, no significant differences were found in terms of age, BMI, FM, diastolic blood pressure, and frequency of MetS between males and females, while normometabolic females showed a significantly higher Tanner stage and hip circumference (*p* < 0.01) and significantly lower waist circumference, BW, height, amount of FFM, systolic blood pressure and frequency of NAFLD (*p* < 0.01).

Both the absolute value of mREE and its adjustment for body weight were significantly lower in normometabolic females compared to those of normometabolic males, of approx. 280 kcal in the first case (*p* < 0.01).

In the hypermetabolic subgroup, no significant differences were found in terms of age, hip circumference, and BMI between males and females, while hypermetabolic females showed a significantly higher Tanner stage (*p* < 0.01) and significantly lower waist circumference, BW, height, amount of both FM and FFM, systolic and diastolic blood pressure and frequency of MetS and NAFLD (*p* < 0.01).

The mREE of hypermetabolic females was also significantly lower than that of hypometabolic males, of approx. 435 kcal. Moreover, for hypermetabolic females, the mREE was significantly lower than eREE (*p* < 0.01) by approx. 312 kcal, and for hypometabolic males of approx. 397 kcal (*p* < 0.01).

Also, in the case of mREE adjusted for body weight, males showed the greatest energy expenditure per kg of body weight compared to females (*p* < 0.01).

When comparing only the female subgroups, no significant differences were found in terms of age, Tanner stage, systolic blood pressure, and frequency of NAFLD in the three metabolic statuses. However, hypermetabolic females showed lower waist and hip circumferences, BW, height, BMI, amount of FM and diastolic blood pressure (*p* < 0.01), and higher amount of FFM and greatest energy expenditure per kg of body weight (*p* < 0.01) compared to the other subgroups, while normometabolic females had the higher frequency of MetS (*p* < 0.01).

Instead, when comparing only the male subgroups, no significant differences were found in terms of waist circumference, height, BMI, systolic and diastolic blood pressure, frequency of both MetS and NAFLD, and eREE in the three metabolic subgroups. However, hypermetabolic males showed a significantly lower Tanner stage, amount of FM (*p* < 0.01), hip circumference, BW and were younger (*p* < 0.05) and had significantly higher amount of FFM and greatest energy expenditure per kg of body weight (*p* < 0.01).

Blood parameters of the three subgroups are shown in Table 3.

**Table 3:** Blood parameters of the three subgroups.

	Hypometabolic (< 90%)	Normometabolic (90%—110%)	Hypermetabolic (> 110%)	*p-* value	*p-*value Hypo vs. Normo	*p-*value Hypo vs. Hyper	*p-*value Hyper vs. Normo
AST (U/L)	21.4 ± 7.1	23.0 ± 9.8	24.3 ± 11.7	**^a^	*^b^	**^b^	ns^b^
ALT (U/L)	25.8 ± 16.5	29.4 ± 23.2	31.0 ± 24.5	*^a^	*^b^	**^b^	ns^b^
GGT (U/L)	17.9 ± 9.9	19.5 ± 13.6	19.1 ± 12.0	ns^a^	ns^b^	ns^b^	ns^b^
ALP (U/L)	172.5 ± 127.0	215.5 ± 165.4	254.3 ± 191.1	**^a^	**^b^	**^b^	**^b^
Bilirubin (mg/dL)	0.7 ± 0.3	0.7 ± 0.3	0.7 ± 0.4	ns^a^	ns^b^	ns^b^	ns^b^
Glucose (mg/dL)	77.9 ± 6.6	78.9 ± 7.5	78.7 ± 8.5	ns^a^	ns^b^	ns^b^	ns^b^
Insulin (µIU/L)	14.8 ± 10.3	14.9 ± 11.3	14.7 ± 8.5	ns^a^	ns^b^	ns^b^	ns^b^
Creatinine (mg/dL)	0.6 ± 0.1	0.6 ± 0.1	0.6 ± 0.1	ns^a^	ns^b^	ns^b^	ns^b^
Blood urea Nitrogen (mg/dL)	26.8 ± 5.2	26.9 ± 5.6	27.3 ± 5.9	ns^a^	ns^b^	ns^b^	ns^b^
Uric acid (mg/dL)	6.0 ± 1.4	6.1 ± 1.3	6.1 ± 1.3	ns^a^	ns^b^	ns^b^	ns^b^
T-C (mg/dl)	161.3 ± 29.9	161.9 ± 29.4	162.3 ± 31.2	ns^a^	ns^b^	ns^b^	ns^b^
HDL-C (mg/dl)	45.2 ± 11.5	43.8 ± 10.2	46.0 ± 11.5	**^a^	ns^b^	ns^b^	**^b^
LDL-C (mg/dl)	102.2 ± 27.2	103.0 ± 26.6	104.0 ± 27.8	ns^a^	ns^b^	ns^b^	ns^b^
VLDL-C (mg/dl)	18.4 ± 8.4	19.0 ± 8.3	19.9 ± 9.2	ns^a^	ns^b^	ns^b^	ns^b^
Triglycerides (mg/dl)	91.5 ± 42.7	95.2 ± 41.7	98.7 ± 44.5	ns^a^	ns^b^	ns^b^	ns^b^
CRP (mg/dl)	0.5 ± 0.5	0.6 ± 0.7	0.6 ± 0.9	ns^a^	ns^b^	ns^b^	ns^b^

*AST* Aspartate transaminase, *ALP* Alanine transaminase, *GGT* Gamma-glutamyl transferase, *ALP* alkaline phosphatase, *T-C* total cholesterol, *HDL-C* HDL cholesterol, *LDL-C* LDL cholesterol, *VLDL-C* VLDL cholesterol, *CRP* C-reactive protein, *ns* non-significant.

*p < 0.05; **p < 0.01.

^a^ANOVA.

^b^T-Student test.

No significant differences were found in terms of GGT, bilirubin, glucose, insulin, creatinine, blood urea nitrogen, uric acid, T-C, LDL-C, VLDL-C, triglycerides and CPR concentrations among the three subgroups. By contrast, the hypometabolic group had a lower concentration of AST and ALT (*p* < 0.01 and* p* < 0.05), these parameters being comparable in the normo- and hypermetabolic groups. The hypermetabolic group had higher concentrations of ALP (*p* < 0.01) and HDL-C compared to the normometabolic (*p* < 0.01), while the hypometabolic had lower ALP concentrations (*p* < 0.01).

Table 4 summarized the comparison of these blood parameters in the three subgroups when further divided into males and females.

**Table 4:** Blood parameters of the three subgroups divided into males and females.

	Hypometabolic			Normometabolic				Hypermetabolic				*p*-value^b^	*p*-value^b^	
	F	M	*p-*value^a^	F		M	*p-*value ^a^	F		M	*p-*value^a^	F	M	
AST (U/L)	19.4 ± 6.3	24.7 ± 7.1	**	20.3 ± 7.5		26.5 ± 11.1	**	22.1 ± 10.5		27.7 ± 12.6	**	**	ns	
ALT (U/L)	21.3 ± 13.5	33.2 ± 18.3	**	22.9 ± 13.8		37.5 ± 29.2	**	25.9 ± 21.0		38.9 ± 27.4	**	*	ns	
GGT (U/L)	15.4 ± 7.8	22.1 ± 11.4	**	16.3 ± 9.1		23.4 ± 16.9	**	17.0 ± 11.7		22.3 ± 12.0	**	ns	ns	
ALP (U/L)	129.2 ± 79.5	242.6 ± 155.9	**	161.4 ± 120.8		282.6 ± 187.3	**	199.1 ± 154.1		339.8 ± 210.9	**	**	**	
Bilirubin (mg/dL)	0.6 ± 0.3	0.7 ± 0.3	ns	0.7 ± 0.3		0.7 ± 0.3	ns	0.7 ± 0.3		0.8 ± 0.4	ns	ns	ns	
Glucose (mg/dL)	76.7 ± 6.5	79.8 ± 6.2	**	78.3 ± 7.7		79.7 ± 7.2	**	78.0 ± 8.6		79.7 ± 8.2	ns	ns	ns	
Insulin (µIU/L)	14.2 ± 9.0	15.8 ± 12.2	**	14.8 ± 12.7		15.1 ± 9.2	ns	13.9 ± 7.6		16.0 ± 9.7	*	ns	ns	
Creatinine (mg/dL)	0.6 ± 0.1	0.7 ± 0.1	ns	0.6 ± 0.1		0.7 ± 0.1	**	0.6 ± 0.1		0.7 ± 0.1	**	ns	ns	
Blood urea Nitrogen (mg/dL)	26.0 ± 5.2	28.1 ± 4.9	**	26.1 ± 5.7		28.0 ± 5.2	**	26.5 ± 5.3		28.5 ± 6.6	**	ns	ns	
Uric acid (mg/dL)	5.5 ± 1.2	6.7 ± 1.5	**	5.8 ± 1.1		6.6 ± 1.3	**	5.8 ± 1.2		6.6 ± 1.4	**	ns	ns	
T-C (mg/dl)	160.0 ± 28.3	163.5 ± 32.5	ns	161.9 ± 28.5		162.0 ± 30.7	ns	164.1 ± 32.7		161.2 ± 28.7	ns	ns	ns	
HDL-C (mg/dl)	47.3 ± 11.1	41.9 ± 11.3	**	44.9 ± 9.8		42.6 ± 10.5	**	47.7 ± 11.5		43.4 ± 11.0	**	**	ns	
LDL-C (mg/dl)	99.7 ± 25.5	106.2 ± 29.3	ns	102.1 ± 26.1		104.2 ± 27.2	ns	103.5 ± 29.3		104.6 ± 25.3	ns	ns	ns	
VLDL-C (mg/dl)	16.8 ± 7.3	21.9 ± 9.4	**	18.7 ± 8.1		19.5 ± 8.6	ns	19.1 ± 9.1		21.2 ± 9.3	*	*	ns	
Triglycerides (mg/dl)	84.4 ± 37.0	103.0 ± 48.7	ns	93.3 ± 40.8		97.5 ± 42.8	ns	95.4 ± 45.6		103.8 ± 42.4	ns	ns	ns	
CRP (mg/dl)	0.5 ± 0.5	0.5 ± 0.4	ns	0.5 ± 0.6		0.6 ± 0.8	ns	0.6 ± 0.9		0.5 ± 0.9	ns	ns	ns	

*M* males, *F* females, *AST* Aspartate transaminase, *ALP* Alanine transaminase, *GGT* Gamma-glutamyl transferase, *ALP* alkaline phosphatase, *T-C* total cholesterol, *HDL-C* HDL cholesterol, *LDL-C* LDL cholesterol, *VLDL-C* VLDL cholesterol, *CRP* C-reactive protein, *ns* non-significant.

*p < 0.05; **p < 0.01.

^a^T-Student test.

^b^ANOVA.

In the hypometabolic subgroup, no significant differences were found in terms of bilirubin, creatinine, T-C, LDL-C, triglycerides and CRP concentrations between males and females. However, hypometabolic females showed significantly higher HDL-C concentration (*p* < 0.01) and lower concentration of all the other parameters, AST, ALT, GGT, ALP, glucose, insulin, blood urea nitrogen, uric acid and VLDL-C (*p* < 0.01).

In the normometabolic subgroup, no significant differences were found in terms of bilirubin, insulin, T-C, LDL-C, VLDL-C, triglycerides and CRP concentrations between males and females, but normometabolic females showed significantly higher HDL-C concentration (*p* < 0.01) and lower concentration of all the other parameters, AST, ALT, GGT, ALP, glucose, creatinine, blood urea nitrogen and uric acid (*p* < 0.01).

In the hypermetabolic subgroup, no significant differences were found in terms of bilirubin, glucose, T-C, LDL-C, triglycerides and CRP concentrations between males and females, but hypermetabolic females showed significantly higher HDL-C concentration (*p* < 0.01) and lower concentration of all the other parameters, AST, ALT, GGT, ALP, creatinine, blood urea nitrogen, uric acid (*p* < 0.01), insulin and VLDL-C (*p* < 0.05).

When comparing the female subgroups, no significant differences were found in terms of GGT, bilirubin, glucose, insulin, creatinine, blood urea nitrogen, uric acid, T-C, LDL-C, triglycerides and CRP concentrations. Hypermetabolic females showed significantly higher concentrations of AST, ALP (*p* < 0.01), ALT and VLDL-C (*p* < 0.01), while normometabolic females had the lowest concentration of HDL-C (*p* < 0.01), which was comparable in the other two subgroup.

When comparing the male subgroups, no significant differences were found in all blood parameters, except for ALP concentration only, which was significantly higher in the hypermetabolic male subgroup (*p* < 0.01) and lowest in the hypometabolic.

The results of the simple linear regression analysis are summarized in Table 5.

**Table 5:** Regression analysis: effect of significant independent variables on mREE in the study population and in each metabolic status.

	Sex	Age	Tanner stage	BW	Height	WC	HC	BMI	NAFLD	MetS	FFM	FM
Population												
Slope	− 324.1	51.29	38.61	12.27	22.68	15.00	15.24	32.23	198.2	252.8	27.76	13.76
Y-intercept	2130	1211	1800	745.2	− 1740	269.1	127.7	758.7	1868	1886	650,2	1241
r squared	0.183	0.06351	0.01866	0.4972	0.3465	0.3347	0.2364	0.2667	0.06587	0.07933	0.4836	0.3178
*p*-value	**	**	**	**	**	**	**	**	**	**	**	**
Hypometabolic												
Slope	− 328.2	55.83	69.87	11.37	20.66	13.92	14.37	30.11	149.9	149.9	26.64	14.15
Y-intercept	1830	823.9	1362	493.9	− 1740	57.31	− 112.7	505.4	1582	1582	411.0	862.8
r squared	0.2963	0.1273	0.1016	0.7667	0.5085	0.5257	0.3837	0.4098	0.05488	0.1701	0.6422	0.613
* p*-value	**	**	**	**	**	**	**	**	**	**	**	**
Normometabolic												
Slope	− 280.5	55.77	50.06	12.78	23.64	15.88	16.93	33.78	169.5	234.1	26.84	14.87
Y-intercept	2075	1124	1734	652,2	− 1933	131.4	− 111.3	667.1	1855	1865	654.1	1146
r squared	0.1886	0.1006	0.04225	0.7617	0.5168	0.5126	0.4166	0.4153	0.06558	0.09466	0.6379	0.5329
* p*-value	**	**	**	**	**	**	**	**	**	**	**	**
Hypermetabolic												
Slope	− 438.4	47.32	11.32	16.77	28.42	19.65	22.21	42.94	201.2	314.3	32.43	20.30
Y-intercept	2436	1497	2128	615,2	− 2397	37.40	− 408.8	639.1	2086	2105	689.2	1215
r squared	0.3042	0.05082	0.001502	0.6744	0.4513	0.4616	0.3396	0.3564	0.06527	0.1067	0.5811	0.4525
* p*-value	**	**	ns	**	**	**	**	**	**	**	**	**
*p*-value ANOVA	ns	*	**	**	**	**	**	**	ns	ns	**	**

*BW* body weight, *WC* waist circumference, *HC* hips circumference, *BMI* body mass index, *NAFLD* non-alcoholic fatty liver disease, *MetS* metabolic syndrome, *FFM* fat-free mass, *FM* fat mass, *ns* non-significant.

**p < 0.01.

The simple regression analysis showed that clinical and anthropometric characteristics influence and are significantly related to metabolism and metabolic status in different manners. In particular, in the whole population, as well as in each subgroup, the male sex and increasing age, BW, height, WH, HC, BMI, FFM and FM positively influence mREE, as well as the presence of NAFLD and MetS. Moreover, the Tanner stage does not have a significant positive effect on mREE only in the hypermetabolic group.

Lastly, the slopes comparison of the variable considered among the three metabolic subgroups showed that only the contribution of sex and presence of NAFLD and Mets was comparable, while there were significant differences in the slopes of the other variables depending on the metabolic status.

## Discussion

For many years, obesity was thought to be associated with an altered energy expenditure compared to that of normal-weight individuals, a situation causing the development and maintenance of the condition of obesity and determining the concept of “slow metabolism”^27^. Some longitudinal studies in the adult population have supported the idea that reduced energy expenditure is a risk factor for obesity development^28^. The same situation was hypothesized to occur in the pediatric population as well, where some studies comparing energy intake among adolescents with obesity and peers with normal-weight highlighted how individuals with obesity seemed to eat similar or even fewer amounts of calories than their normal weighted peers^29,30^. Since obesity is caused by an imbalance between energy intake and expenditure, i.e., when intake exceeds energy consumption^31^, it was hypothesized that weight gain could be attributed to an altered metabolism (i.e., a reduction in REE resulting in the development of the condition despite low or adequate energy intakes^30^). By contrast, this conclusion was disproved by the fact that other studies suggested that people with obesity might have higher REE compared to normal-weight subjects, both in the adult and pediatric population^27,30,32–34^.

Taking into account these conflicting results, one of the aims of the present research was to assess the actual metabolic condition of the population in pediatric age with obesity.

In the present study, according to what has already been reported by our group for the adult population with obesity^26^, only a minor portion of the pediatric population recruited showed a reduced REE, while the major part was normo- and hypermetabolic. The prevalence of the hypometabolic condition in our pediatric population with obesity (13.9%) was slightly higher than that observed in our adult population with obesity (8%)^26^, thus confirming that a reduced basal metabolism is not commonly implicated, as previously believed, in causing and maintaining obesity. This is especially significant considering that, the majority of the participants were actually normo- or even hypermetabolic and had obesity anyway.

In this regard, it is worth noting how, in our study population, the potential low-calorie diet prescribed based on the eREE would have determined a smaller caloric restriction in the hypometabolic subgroup than that actually required for the patient, resulting in a slighter body weight reduction, and, by contrast, a severe caloric restriction in the hypermetabolic subgroup, probably difficult to be tolerated and maintained for a prolonged period.

As far as the analysis of the three subgroups, when considering body composition, hypermetabolic participants with obesity were significantly lighter in terms of both BW and BMI, slimmer in terms of body circumferences, and had a greater amount of FFM. The FFM compartment, which predominantly consists of muscles, bones, and water, is considered one of the most active metabolic tissues of the body, and as such, it is a significant factor in determining mREE^14,30^. In fact, the hypermetabolic group showed a greater energy expenditure per kg of body weight, probably due to their greater amount of FFM. Unfortunately, the level of physical activity of the participants was not investigated in the present study, and, for this reason, it is not possible to conclude whether exercise was a determinant for these characteristics in this metabolic status.

Furthermore, despite the fact that gender is a significant factor in determining REE and metabolic status variability^34^ (i.e., absolute REE values are higher in males than in females), no significant gender-related differences were observed in the prevalence of the three metabolic statuses.

However, gender differences appeared when the metabolic subgroups were divided into males and females. In fact, within the same subgroup, females are generally shorter, lighter (lower BW), and slimmer (with lower WC and HC) than males with the same BMI, resulting in lower energy expenditure in terms of both absolute and per kg of body weight. Comparing the female and male populations between the different subgroups, the hypermetabolic group consistently exhibits the best anthropometric and clinical characteristics.

Even analyzing the different blood parameters, within the same metabolic group, females have a better condition, with lower concentrations of liver transaminases, a lower frequency of NAFLD, and a better lipid profile, characteristics which remain constant in the three subgroups.

Age has been considered a determinant of REE, being correlated negatively in adults and positively in children and adolescents^35–37^. In the present study, age in the three subgroups and between males and females was however comparable.

Interestingly, there were differences in the frequency of NAFLD. In other studies, low and/or high levels of mREE have been associated with various comorbidities including metabolic syndrome, insulin resistance and NAFLD^38^. In fact, the presence of inflammatory diseases, including NAFLD, was shown to increase mREE^39^, since, inflammation is associated with an elevated VO_2_, enhanced lipolysis, high concentration of catabolic hormones, extensive protein catabolism, and maintaining the immune function case an increase of approx. 15% of daily energy expenditure^39–41^. For this reason, REE predictive equations were demonstrated to be less accurate in case of liver impairment, causing an underestimation of REE^42–46^. The results of the present study showed that while the presence of MetS was not related to the metabolic state since it was comparable in the three subgroups, prevalence of NAFLD was linked with higher mREE values and its incidence was significantly higher in the hypermetabolic subgroup and in males.

Lastly, the regression analysis highlighted that all the independent variables considered significantly influence metabolism and the presence of significant differences in some of the slopes among the three groups indicate that their contribution to the definition of mREE varied according to the metabolic status.

In conclusion, our data show that obesity in children and adolescents is associated more with a normal or an increased REE than with a hypometabolic condition, suggesting that reduced energy expenditure is not the main cause of obesity in children and adolescents as well as in adults. Moreover, although the existence and the effort to adapt and validate valuable and accurate equations to estimate REE, the present study further highlights how they are unsuitable for the population with obesity, especially in the presence of comorbidities such as NAFLD. In fact, the Molnar equation, which is thought to be the most accurate for children and adolescents with obesity^21,25^, wrongly predicted REE in approx. 40% of the patient recruited, with either an under or an overestimation. This percentage is markedly high and further stresses the need in the clinical practice for a precise REE assessment to ensure adequate caloric intake, especially in children and adolescents with severe obesity. The risk of inappropriate caloric targets when using predictive equations remains sufficiently great to suggest that indirect calorimetry, when available, should be preferred in order to prevent under- or over-feeding and their related consequences, which might be important in this critical period of growth and pubertal development. Some consequences of inadequate intakes are too rapid weight loss (for an excessive lower intake), causing malnutrition and FFM depletion, especially on skeletal muscle mass and function (causing sarcopenia), and increased risk of withdrawal or, by contrast (for a smaller reduction of energy intake), poor weight loss or weight stasis or gain.

Therefore, inadequate diet therapies based on eREE might be one of the reasons explaining the failure of current interventions to achieve any meaningful and long-term results and the high frequency of failure and drop-out of weight loss programs in the pediatric population with obesity, which ultimately determines the persistence of the condition in adulthood. For this reason, in order to actively counteract the increasing obesity epidemic among children and adolescents, it is imperative to develop individualized, precise, and effective therapies, possibly with a multidisciplinary approach, associating to the adequate and tailored diet plan also physical activity and behavioral intervention.

## Methods

### Study population

One-thousand and four hundred children and adolescents with obesity were recruited at the Division of Auxology, Istituto Auxologico Italiano, IRCCS, Piancavallo (VB), where they were hospitalized for a 3-week multidisciplinary body weight reduction program (BWRP). The inclusion criteria were: i. individuals of both sexes, aged between 10 and 18 years, ii. BMI > 97th percentile from age and sex using the Italian reference curves^47^, iii. absence of any concomitant drug treatment known to interfere with REE evaluation (i.e., beta-blockers, thyroid hormones, anti-depressants, etc).

The study was approved by the Ethical Committee of Istituto Auxologico Italiano, Milan, Italy (research code: 2022_03_15_05, acronym: REEOBPED). The purpose and objective of the study were explained in detail to each subject and their parents and written informed consent was obtained before the beginning of the study.

### REE measurement and REE estimation

At the beginning of the BWRP, mREE was measured between 8.00 and 10.00 AM, in thermo-neutral conditions (room temperature: 22°–25 °C) using an open-circuit indirect computerized calorimeter equipped with a canopy (Vmax 29, Sensor Medics, Yorba Linda, CA), periodically undergone to quality control tests in order to ensure the reliability of the measurements. The gas analyzers were calibrated before each test using a reference gas mixture made of 15% O_2_ and 5% CO_2_. The participants were fasting from at least 8 h, were not smoking for at least 1 h and waited 30 min in a sitting position before undergoing REE measurement. mREE was assessed in the supine position for at least 30 min, including an acclimation period of 10 min. The data relative to the acclimation period were discarded. The steady state was defined as at least 5 min less than 5% variation in the respiratory quotient, less than 10% variation in O_2_ and less than 10% variation in minute ventilation^48^. After the steady state was reached, O_2_ consumption and CO_2_ production were recorded at intervals of 1 min for at least 20 min and averaged over the whole measurement period. REE was calculated from O_2_ consumption and CO_2_ production using Weir’s equation^49^.

eREE was estimated using the Molnar equation^25^ as follows:

$${\text{eREE Males}} = \left(50.9 \times {\text{weight }}\left[ {{\text{kg}}} \right] \right) + \left( 25.3 \times {\text{height }}\left[ {{\text{cm}}} \right] \right) - \left( 50.3 \times {\text{age }}\left[ {{\text{years}}} \right] \right) + 26.9,$$

$${\text{eREE Females}} = \left( 51.2 \times {\text{weight }}\left[ {{\text{kg}}} \right] \right) + \left( 24.5 \times {\text{height }}\left[ {{\text{cm}}} \right] \right) - \left( 207.5 \times {\text{age }}\left[ {{\text{years}}} \right] \right) + 1629.8.$$

### Anthropometry

Body weight (BW) and height were measured at the time of admission to the hospital following international guidelines^50^ using a scale with a stadiometer (Wunder Sa.Bi., WU150, Trezzo sull’Adda, Italy) with the subject only wearing underclothes. BMI was calculated as weight (kg)/height (m)^2^.

Body composition was measured at the beginning of the BWRP by using a multifrequency tetrapolar impedancemeter (BIA, Human-IM Scan, DS-Medigroup, Milan, Italy) with a delivered current of 800 μA at a frequency of 50 kHz. In order to reduce errors of measurement, special care was paid to the standardization of the variables known to affect measurement validity, reproducibility, and precision. Measurements were performed according to the method of Lukaski^51^ (after 20 min resting in a supine position with arms and legs relaxed and not in contact with other body parts) and in strictly controlled conditions.

Waist (WC) was measured at the midpoint between the last rib and the iliac crest and hip circumference (HC) was measured at the largest parts around the buttocks using a flexible tape measure.

### Laboratory and clinical measurements

Blood samples (about 10 mL) were collected early in the morning after an overnight fast in standard tubes for serum. Concentrations of aspartate transaminase (AST), alanine transaminase (ALT), gamma-glutamyl transferase (GGT), alkaline phosphatase (ALP), bilirubin, glucose, insulin, creatinine, blood urea nitrogen, uric acid, total (T-C), HDL (HDL-C), LDL (LDL-C) and VLDL (VLDL-C) cholesterol, triglycerides and C-reactive protein (CRP) were measured by the same internal laboratory using standard methods.

Systolic (SBP) and diastolic blood pressure (DBP) were measured twice (3-min intervals in-between) on the dominant arm with an aneroid sphygmomanometer (TemaCertus, Milan, Italy), by using appropriate-sized cuffs for young participants with obesity. The mean values were calculated and rounded to the nearest 5 mmHg value.

The presence of metabolic syndrome (MetS) was determined according to the IDF (International Diabetes Federation) criteria for diagnosis in children and adolescents^52^. In particular, MetS was defined in the presence of: WC ≥ 90th percentile for ages < 16 years, and ≥ 94 cm for males and ≥ 80 cm for females aged > 16 years, plus two or more of the following factors: i. TG concentration: ≥ 150 mg/dL or in pharmacological treatment for dyslipidemia; ii. HDL-C: < 40 mg/dL for males and females for ages < 16 years, and < 40 mg/dL for males and < 50 mg/dL for females, or in pharmacological treatment for dyslipidemia; iii. SBP ≥ 130 mmHg or DBP ≥ 85 mmHg; iv. fasting glycemia ≥ 100 mg/dL or diagnosis of type 2 diabetes mellitus.

The diagnosis of non-alcoholic fatty liver disease (NAFLD) was made by the same expert echographist through accurate liver ultrasonography using standard criteria^53,54^, as previously described^55^.

### Statistical analysis

Continuous variables are expressed as means ± standard deviation, and categorical variables as absolute and relative frequency. The Shapiro–Wilk test showed that all parameters were normally distributed.

The study population was divided into three subgroups based on the percentage ratio of mREE and eREE predicted by the Molnar equation, as described. Moreover, each subgroup was further divided into males and females.

All parameters were compared among these three subgroups and between males and females by using the appropriate test, in particular, the t-Student test, ordinary one-way ANOVA, Chi squared test, or Fisher’s exact test.

Simple linear regression analysis for mREE (dependent variable) was performed using as possible single independent variables sex (as dummy variable as male = 0 and female = 1), age, Tanner stage, BW, height, WC, HC, BMI, presence of NAFLD and Mets (both as dummy variables as no = 0 and yes = 1), FFM, and FM. The analysis was performed on the whole population and the three subgroups to define the effect of the selected variable in each metabolic status. A comparison of regression parameters, in particular slopes, was used to underline significant differences.

A level of significance of *p* < 0.05 was used for all data analysis.

### Institutional review board statement

The study was conducted in accordance with the Declaration of Helsinki, and approved by the Ethics Committee of Istituto Auxologico Italiano (research code: 2022_03_15_05, acronym: REEOBPED).

### Informed consent statement

Written informed consent was obtained from participants and their parents.

## Data Availability

The dataset generated and analyzed in the present study are available from the corresponding author on reasonable request.

## References

[1] Di Cesare M (2019). The epidemiological burden of obesity in childhood: A worldwide epidemic requiring urgent action. BMC Med..

[2] Jebeile H, Kelly AS, O’Malley G, Baur LA (2022). Obesity in children and adolescents: Epidemiology, causes, assessment, and management. Lancet Diabetes Endocrinol..

[3] Llewellyn A, Simmonds M, Owen CG, Woolacott N (2016). Childhood obesity as a predictor of morbidity in adulthood: A systematic review and meta-analysis. Obes Rev. Off. J. Int. Assoc. Study Obes..

[4] Simmonds M, Llewellyn A, Owen CG, Woolacott N (2016). Predicting adult obesity from childhood obesity: A systematic review and meta-analysis. Obes. Rev..

[5] Storz MA (2020). The COVID-19 pandemic: An unprecedented tragedy in the battle against childhood obesity. Clin. Exp. Pediatr..

[6] Kansra AR, Lakkunarajah S, Jay MS (2021). Childhood and adolescent obesity: A review. Front. Pediatr..

[7] Romieu I (2017). Energy balance and obesity: What are the main drivers?. Cancer Causes Control.

[8] Dabas A, Seth A (2018). Prevention and management of childhood obesity. Indian J. Pediatr..

[9] Tremblay MS, Colley RC, Saunders TJ, Healy GN, Owen N (2010). Physiological and health implications of a sedentary lifestyle. Appl. Physiol. Nutr. Metab. Physiol. Appl. Nutr. Metab..

[10] González-Gross M, Meléndez A (2013). Sedentarism, active lifestyle and sport: Impact on health and obesity prevention. Nutr. Hosp..

[11] Rigamonti AE (2020). Impact of a three-week in-hospital multidisciplinary body weight reduction program on body composition, muscle performance and fatigue in a pediatric obese population with or without metabolic syndrome. Nutrients.

[12] Lazzer S (2022). Effects of a 3-Week inpatient multidisciplinary body weight reduction program on body composition and physical capabilities in adolescents and adults with obesity. Front. Nutr..

[13] Smout MF (2022). Pediatric quality of life multidimensional fatigue scale (PedsQL-MFS) detects the effects of a 3-week Inpatient body weight reduction program for children and adolescents with obesity. Health Qual. Life Outcomes.

[14] Goran MI, Treuth MS (2001). Energy expenditure, physical activity, and obesity in children. Pediatr. Clin. North Am..

[15] Goran MI (1995). Variation in total energy expenditure in humans. Obes. Res..

[16] Müller MJ, Bosy-Westphal A (2003). Assessment of energy expenditure in children and adolescents. Curr. Opin. Clin. Nutr. Metab. Care.

[17] Kohorst MA, Warad DM, Nageswara Rao AA, Rodriguez V (2018). Obesity, sedentary lifestyle, and video games: The new thrombophilia cocktail in adolescents. Pediatr. Blood Cancer.

[18] Rodríguez-Núñez I, Valderrama Erazo P (2021). Sedentary lifestyle and obesity in pediatrics: the other pandemic. Andes Pediatr. Rev. Chil. Pediatr..

[19] Lam YY, Ravussin E (2017). Indirect calorimetry: An indispensable tool to understand and predict obesity. Eur. J. Clin. Nutr..

[20] Madden AM, Mulrooney HM, Shah S (2016). Estimation of energy expenditure using prediction equations in overweight and obese adults: A systematic review. J. Hum. Nutr. Diet. Off. J. Br. Diet. Assoc..

[21] Bedogni G (2020). External validation of equations to estimate resting energy expenditure in 2037 children and adolescents with and 389 without obesity: A cross-sectional study. Nutrients.

[22] Bedogni G (2019). External validation of equations to estimate resting energy expenditure in 14952 adults with overweight and obesity and 1948 adults with normal weight from Italy. Clin. Nutr. Edinb. Scotl..

[23] Mifflin MD (1990). A new predictive equation for resting energy expenditure in healthy individuals. Am. J. Clin. Nutr..

[24] Itani L, Tannir H, Kreidieh D, El Masri D, El Ghoch M (2020). Validation of predictive equations for resting energy expenditure in treatment-seeking adults with overweight and obesity: Measured versus estimated. J. Popul. Ther. Clin. Pharmacol..

[25] Molnár D, Jeges S, Erhardt E, Schutz Y (1995). Measured and predicted resting metabolic rate in obese and nonobese adolescents. J. Pediatr..

[26] Tamini S, Cicolini S, Caroli D, Sartorio A (2021). Effects of a 3-week in-hospital multidisciplinary body weight reduction program in obese females: Is measured resting energy expenditure essential for tailoring adequately the amount of energy intake?. Front. Nutr..

[27] Cacciari E (2006). Italian cross-sectional growth charts for height, weight and BMI (2 to 20 year). J. Endocrinol. Invest..

[28] McClave SA (2003). Clinical use of the respiratory quotient obtained from indirect calorimetry. JPEN J. Parenter. Enteral Nutr..

[29] Weir JBDB (1949). New methods for calculating metabolic rate with special reference to protein metabolism. J. Physiol..

[30] Lohman TG, Roche AF, Martorell R (1991). Anthropometric Standardization Reference Manual.

[31] Lukaski HC (1987). Methods for the assessment of human body composition: Traditional and new. Am. J. Clin. Nutr..

[32] Zimmet P (2007). The metabolic syndrome in children and adolescents—An IDF consensus report. Pediatr. Diabetes.

[33] Saverymuttu SH, Joseph AE, Maxwell JD (1986). Ultrasound scanning in the detection of hepatic fibrosis and steatosis. Br. Med. J. Clin. Res. Ed..

[34] Sartorio A (2007). Predictors of non-alcoholic fatty liver disease in obese children. Eur. J. Clin. Nutr..

[35] Bedogni G (2021). Development and internal validation of fatty liver prediction models in obese children and adolescents. J. Clin. Med..

[36] Schober P, Boer C, Schwarte LA (2018). Correlation coefficients: Appropriate use and interpretation. Anesth. Analg..

[37] Carneiro IP (2016). Is obesity associated with altered energy expenditure?. Adv. Nutr..

[38] Goran MI (2000). Energy metabolism and obesity. Med. Clin. North Am..

[39] Stefanik PA, Heald FP, Mayer J (1959). Caloric intake in relation to energy output of obese and non-obese adolescent boys. Am. J. Clin. Nutr..

[40] Bandini LG, Schoeller DA, Dietz WH (1990). Energy expenditure in obese and nonobese adolescents. Pediatr. Res..

[41] Hopkins M, Blundell JE (2016). Energy balance, body composition, sedentariness and appetite regulation: Pathways to obesity. Clin. Sci..

[42] Elbelt U (2010). Differences of energy expenditure and physical activity patterns in subjects with various degrees of obesity. Clin. Nutr. Edinb. Scotl..

[43] DeLany JP, Kelley DE, Hames KC, Jakicic JM, Goodpaster BH (2013). High energy expenditure masks low physical activity in obesity. Int. J. Obes..

[44] Zapata JK (2021). Resting energy expenditure is not altered in children and adolescents with obesity. Effect of age and gender and association with serum leptin levels. Nutrients.

[45] Molnár D, Schutz Y (1997). The effect of obesity, age, puberty and gender on resting metabolic rate in children and adolescents. Eur. J. Pediatr..

[46] Maffeis C, Schutz Y, Micciolo R, Zoccante L, Pinelli L (1993). Resting metabolic rate in six- to ten-year-old obese and nonobese children. J. Pediatr..

[47] Roberts SB, Rosenberg I (2006). Nutrition and aging: Changes in the regulation of energy metabolism with aging. Physiol. Rev..

[48] Hosseini B, Mirzaei K, Maghbooli Z, Keshavarz SA, Hossein-Nezhad A (2016). Compare the resting metabolic rate status in the healthy metabolically obese with the unhealthy metabolically obese participants. J. Nutr. Intermed. Metab..

[49] Reddavide R (2019). Non-alcoholic fatty liver disease is associated with higher metabolic expenditure in overweight and obese subjects: A case-control study. Nutrients.

[50] Simone U (2005). Inflammation is associated with increased energy expenditure in patients with chronic kidney disease. Am. J. Clin. Nutr..

[51] Buttgereit F, Burmester GR, Brand MD (2000). Bioenergetics of immune functions: Fundamental and therapeutic aspects. Immunol. Today.

[52] Martincevic I, Mouzaki M (2018). Using an allometric equation to accurately predict the energy expenditure of children and adolescents with nonalcoholic fatty liver disease. JPEN J. Parenter. Enteral Nutr..

[53] Carpenter A, Ng VL, Chapman K, Ling SC, Mouzaki M (2017). Predictive equations are inaccurate in the estimation of the resting energy expenditure of children with end-stage liver disease. JPEN J. Parenter. Enteral Nutr..

[54] Oliveira A, Fernandes SA, Carteri RB, Tovo CV (2021). Evaluation of rest energy expenditure in patients with non alcoholic fatty liver disease. Arq. Gastroenterol..

[55] Martincevic I, Mouzaki M (2017). Resting energy expenditure of children and adolescents with nonalcoholic fatty liver disease. J. Parenter. Enter. Nutr..

